# Evaluation of Blood Regimen on the Survival of *Cimex lectularius* L. Using Life Table Parameters

**DOI:** 10.3390/insects4020273

**Published:** 2013-06-18

**Authors:** Alexis M Barbarin, Ron Gebhardtsbauer, Edwin G. Rajotte

**Affiliations:** 1 Department of Entomology, The Pennsylvania State University, 501 ASI Building, University Park, PA 16802, USA; E-Mail: egrajotte@psu.edu; 2 Smeal College of Business, Department of Risk Management (Actuarial Science), The Pennsylvania State University, 331 Business Building, University Park, PA 16802, USA; E-Mail: rug16@psu.edu

**Keywords:** *Cimex lectularius*, life table, survival, rabbit blood, human blood

## Abstract

Knowledge of bed bug development under varying conditions can lead to more sophisticated management techniques. Development rate, age and stage-specific life tables were compared for a laboratory strain (HS) and field strain (ECL-05) of bed bug *Cimex lectularius* L. (Hemiptera: Heteroptera) reared on two blood regimens: human or rabbit blood. Harlan and ECL-05 bed bugs reared on human blood had a life expectancy of 207 and 208 days respectively from the egg stage. Egg to adult development of HS bed bugs reared on human blood (~35 days) was significantly longer than that of the ECL-05 strain (~33 days) in the third, fourth, and fifth instars. The HS and ECL-05 bed bugs reared on rabbit blood had a life expectancy of 149 and 174 days respectively. Egg to adult development time of HS on rabbit blood (~52 days) was significantly longer than ECL-05 (~37 days) in every instar, and HS total life span was significantly shorter compared to ECL-05. Developmental differences based on strain and blood regimen suggest rabbit blood is an inferior blood source for colony maintenance, and strain has variable effects on bed bug development. Findings suggest that blood regimen should strongly be considered in bed bug colony maintenance.

## 1. Introduction

Infestations of the common bed bug, *Cimex lectularius*, have been on the rise across the United States, Canada, Europe, Asia, Africa and Australia over the past ten years [1,2,3,4,5,6]. Despite being closely associated with humans throughout recorded time [7,8], much of the basic behavior and biology of bed bugs is poorly understood, garnering increased attention from researchers throughout the world. Unlike other hematophagous insects, including mosquitoes, tsetse flies, lice, fleas, and assassin bugs, bed bugs are not known to vector any human pathogen [8,9]. However, their presence is known to create quality-of-life concerns [7] including allergic reactions to bites, secondary infections, anxiety, insomnia, and deteriorating mental health conditions [10,11,12].

Bed bugs have five immature stages prior to reaching adulthood, and a blood meal is required for growth and development through each stage. Wild bed bug populations have been known to feed on the blood of several species of birds [13,14] and bats [15,16,17]. In the laboratory, the bed bug has been successfully reared on chicken blood [18,19,20], guinea pig blood [21,22], and rabbit blood [8,23,24]. 

Previous studies evaluated the life history of bed bugs using life tables, [25,26], but only one has evaluated survival based on diets of human, mouse, and hen blood [27]. While those results indicate that the development of bed bugs on alternative blood sources does not significantly depart from development on its traditional human host, the most obvious differences were witnessed among those bed bugs reared on mice [27]. However, as these experiments were conducted prior to the onset of widespread pesticide resistance in bed bugs, it is unlikely that the bed bug populations of today are unchanged from those of the 1930’s [26]. Additionally, while many hematophagous insects will feed on alternative hosts in the absence of the primary host, there are often physiological consequences associated with feeding on unusual host species [28].

The success of blood sucking insects depends on its ability to locate and specialize on a particular host or set of hosts [28]. Reduced fecundity can manifest in many ways including reduced developmental rates, decreased longevity, skewed sex ratio, feeding reduction, and a decreased rate of digestion [29,30,31]. In many cases, the exact components that make the blood of one host beneficial and another detrimental are largely unclear [28]. Blood supplies nutrients, but can also harbor host immune hormones or parasites that impair insect development and must be overcome.

Research illustrates that the tropical bed bug *Cimex hemipterus* performs best on human blood compared to the blood of rabbits, rats, chickens, and bulbuls [32]. While human blood is probably the optimal host blood of *Cimex lectularius*, it is both costly and difficult to obtain for bed bug research, driving many researchers to use substitutes that may be inferior. Recent publications in bed bug research have used non-human blood to maintain bed bug colonies, and have inferred life history parameters, pesticide reactions, behavior and other biological attributes without taking blood regimen into account. If there are systematic differences in bed bugs reared on different species’ blood, then conclusions drawn from these studies must be adjusted. 

Actuaries, demographers, economists, public health professionals and biological scientists have employed life tables to illustrate the life history characteristics of populations over time [33]. Life tables can measure population mortality, survivorship, life expectation, and fecundity [34]. Cohort tables in particular provide a longitudinal perspective of a particular cohort from birth to death, and are frequently used in laboratory studies to illustrate the life history of short-lived species [35].

While several studies have used life tables to compare bed bug populations based on strain [26], none have investigated the life expectancy of bed bugs based on blood regimen. This study, the first of its kind, aims to evaluate the development and life expectancy of two strains of human bed bug reared on either human or rabbit blood [36].

## 2. Materials and Methods

### 2.1. Bed Bugs

A pyrethroid susceptible laboratory strain of bed bugs was obtained in June 2007 from Virginia Polytechnic Institute and State University, Blacksburg, Virginia. This strain was originally established by Harold Harlan (National Pest Management Association, Fairfax, VA, USA) from a population collected in Fort Dix, NJ, USA, and has been restricted to the laboratory, without wild type introductions since 1973. This strain is designated as “HS”. 

A second strain was obtained from the University of Minnesota, St. Paul, Minnesota, USA in November 2010. This strain was established by Ecolab^®^ employees in 2005, and is comprised of specimens collected from Minnesota, Wisconsin, Florida, and New Jersey. This strain has been exposed to pesticide pressure, multiple genetic introductions and other forces encountered in the wild, and will be designated as “ECL-05”.

Colonies of both strains were maintained in Headhouse III at the Pennsylvania State University in University Park, PA, USA. Both strains were reared under standard environmental conditions of 27 ± 0.5 °C, 50 ± 5% relative humidity (RH), and 14:10 (L:D) photoperiod in glass jars containing folded filter paper (Whatman No1, 90 mm) for a harborage. Environmental conditions were selected to ensure maximum survival and fecundity [37]. Jars were covered with organza fabric with a pore size <1 mm for ventilation. Individuals from both colonies were offered a human blood meal weekly via an artificial feeding system [22]. 

### 2.2. Egg Collection

Life table parameters were derived starting with newly oviposited eggs. Fifth-instar bed bug nymphs from each strain were removed from the main colony and placed into separate rearing jars based on instar. Insects were allowed to feed on human blood via an artificial feeding system [22] and were followed to adulthood. Adults were sustained on the same blood type as their progeny; those adults producing eggs for the human blood reared treatment were fed human blood; adults producing eggs to be reared on rabbit blood were fed rabbit blood. 

### 2.3. Blood Feeding and Rearing

Once thirty adults of each sex were available for each treatment, 15 males and 15 females were randomly selected and allowed to mate. One hundred eggs were randomly selected for each blood regimen; fifty bed bugs of each strain were reared on human blood; fifty bed bugs of both strains were reared on rabbit blood. Bed bugs were placed individually into 30 mL plastic diet cups (Dart, USA) and observed daily from egg to death. Immediately after hatching, first instar bed bugs in the human blood treatment were allowed to feed on a human volunteer (IRB# 36511) every second day to ensure adequate access to food was not a limiting factor in the immature stages. Individuals in the rabbit blood treatment were reared on defibrinated rabbit blood (Hemostat Laboratories, Dixon, CA, USA) every second day via an artificial feeding system [22] during the immature stages using a Parafilm^®^ M Barrier film membrane. The Parafilm^®^ was rubbed on the skin of a human participant to provide phagostimulation, and then placed onto the feeder. 

### 2.4. Abridged Life Table Construction

Eggs and subsequent nymphs were observed daily until death. Presence of exuviae in diet cups signified the beginning of a new stadium. Data were collected daily on the number of live and dead individuals in both human blood and rabbit blood fed HS and ECL-05 bed bugs. Data on development and survival were used to construct abridged cohort life tables using the parameters illustrated in Birch [38], Carey [39], and Siegel and Swanson [33]. Mean time in instar was calculated using methods outlined in Carey [39].

### 2.5. Data Analysis

All data were analyzed using SPSS (Version 18.0. Chicago: SPSS Inc.). Data were checked for normality prior to analysis. Transformations of data did not meet the assumption of normality, therefore a non-parametric test (Mann-Whitney U) was used to determine differences in development of each strain and blood regimen based on instar. Further, total survival time and differences in male and female mean survival were assessed using Kaplan-Meier survival analysis.

## 3. Results

### 3.1. Abridged Cohort Table of Human Blood Fed Bed Bugs

Life tables illustrate the expected number of additional days to be lived by a member of the cohort surviving to age *x*. The survivorship of both HS and ECL-05 strains of *Cimex lectularius* are illustrated in abridged form in Table 1, Table 2. While the HS bed bugs reared on human blood have a life expectancy (*e_x_*) of 207 days in the egg stage, ECL-05 bed bugs reared on human blood have an *e_x_* of 208 days. HS and ECL-05 bed bugs reared on human blood had a 98% and 100% chance of surviving to the adult stage respectively.

**Table 1:** Stage specific life table of Harlan strain *Cimex lectularius* on human blood.

Harlan Strain reared on Human Blood									
Stage (n = duration in days)	*N_x_*	Age Interval (*x*)	(*l_x_*)	(*_n_p_x_*)	(*_n_q_x_*)	(*_n_d_x_*)	(*_n_L_x_*)	(*T_x_*)	(*e_x_*)
Egg (n = 6)	50	0–6	1.00	1.00	0.00	0.00	0.00	207.11	207.11
First Instar (n = 6)	50	6–12	1.00	0.98	0.02	0.02	5.94	201.11	201.11
Second Instar (n = 5)	49	12–17	0.98	1.00	0.00	0.00	4.90	195.17	199.15
Third Instar (n = 5)	49	17–22	0.98	1.00	0.00	0.00	4.90	190.27	194.15
Fourth Instar (n = 6)	49	22–28	0.98	1.00	0.00	0.00	5.88	185.37	189.15
Fifth Instar (n = 7)	49	28–35	0.98	1.00	0.00	0.00	6.86	179.49	183.15
Adult (n = 40)	49	35–75	0.98	0.98	0.02	0.02	38.80	172.63	176.15
Adult (n = 40)	48	75–115	0.96	0.96	0.04	0.04	37.60	133.83	139.41
Adult (n = 40)	46	115–155	0.92	0.96	0.04	0.04	36.00	96.23	104.60
Adult (n = 40)	44	155–195	0.88	0.84	0.16	0.14	32.40	60.23	68.44
Adult (n = 40)	37	195–235	0.74	0.35	0.65	0.48	20.00	27.83	37.61
Adult (n = 40)	13	235–275	0.26	0.38	0.62	0.16	7.20	7.83	30.12
Adult (n = 10)	5	275–285	0.10	0.20	0.80	0.08	0.60	0.63	6.30
Adult (n = 3)	1	285–288	0.02	0.00	1.00	0.02	0.03	0.03	1.50

* Notes: *x* is age interval in days, *N* is the number of individuals alive at the beginning of age *x*, *l_x_* is the probability of surviving to age *x*, *_n_q_x_* is the proportion of individuals alive at the onset of the interval, age x that die prior to reaching the end of the interval (*x* + *n*) where n is the duration spent in the interval, *_n_d_x_* is the fraction of the original cohort that die between the interval (*x* to *x* + *n*), *_n_L_x_* is the fraction of interval lived during the designated interval (*x* to *x* + *n*) per individual, *T_x_* is the total number of days lived beyond age (*x*), and *e_x_* is the life expectancy of an individual alive at age *x*.

**Table 2:** Stage specific life table of (field strain) ECL-05 strain *Cimex lectularius* on human blood.

ECL-05 Strain reared on Human Blood									
Stage (n = duration in days)	*N_x_*	Age Interval (*x*)	(*l_x_*)	(*_n_p_x_*)	(*_n_q_x_*)	(*_n_d_x_*)	(*_n_L_x_*)	(*T_x_*)	(*e_x_*)
Eggs (n = 7)	50	0–7	1.00	1.00	0.00	0.00	7.00	208.05	208.05
First Instar (n = 5)	50	7–12	1.00	1.00	0.00	0.00	5.00	201.05	201.05
Second Instar (n = 5)	50	12–17	1.00	1.00	0.00	0.00	5.00	196.05	196.05
Third Instar (n = 5)	50	17–22	1.00	1.00	0.00	0.00	5.00	191.05	191.05
Fourth Instar (n = 5)	50	22–27	1.00	1.00	0.00	0.00	5.00	186.05	186.05
Fifth Instar (n = 6)	50	27–33	1.00	1.00	0.00	0.00	6.00	181.05	181.05
Adult (n = 40)	49	33–73	1.00	0.98	0.02	0.02	39.60	175.05	175.05
Adult (n = 40)	46	73–113	0.98	0.94	0.06	0.06	38.00	135.45	138.21
Adult (n = 40)	46	113–153	0.92	0.93	0.07	0.06	35.60	97.45	105.92
Adult (n = 40)	43	153–193	0.86	0.77	0.23	0.20	30.40	61.85	71.92
Adult (n = 40)	33	193–233	0.66	0.58	0.42	0.28	20.80	31.45	47.65
Adult (n = 40)	19	233–273	0.38	0.26	0.74	0.28	9.60	10.65	28.03
Adult (n = 20)	5	273–293	0.10	0.00	1.00	0.10	1.05	1.05	10.50

* Notes: *x* is age interval in days, *N* is the number of individuals alive at the beginning of age *x*, *l_x_* is the probability of surviving to age *x*, *_n_q_x_* is the proportion of individuals alive at the onset of the interval, age *x* that die prior to reaching the end of the interval (*x* + *n*), where n is the duration spent in the interval, *_n_d_x_* is the fraction of the original cohort that die between the interval (*x* to *x* + *n*), *_n_L_x_* is the fraction of interval lived during the designated interval (*x* to *x* + *n*) per individual, *T_x_* is the total number of days lived beyond age (*x*), and *e_x_* is the life expectancy of an individual alive at age *x*.

Mann Whitney U tests were used to calculate the average time spent in each instar based on strain. HS individuals spent significantly more time in the third (5.02 ± 0.165 days , *p* = 0.001), fourth (5.42 ± 0.190 days, *p* ≤ 0.000), and fifth instars (7.36 ± 0.284 days , *p* ≤ 0.000); ECL-05 individuals spent 4.52 ± 0.119 days, 4.74 ± 0.124 days, and 6.30 ± 0.146 days in third, fourth, and fifth instars respectively (Table 3). However, when comparing the total life span of HS and ECL-05 using Kaplan-Meier Analysis, there were no significant differences (Chi square = 0.382, *p* = 0.536).

**Table 3:** Mean development time (in days) of human blood reared Harlan and ECL-05 strains.

Life Stage	Strain	Mean ± S.E.	*p*
Egg	Harlan	6.40 ± 0.125	=0.325
	ECL-05	6.56 ± 0.128	
First Instar	Harlan	5.56 ± 0.261	=0.115
	ECL-05	5.02 ± 0.168	
Second Instar	Harlan	5.14 ± 0.206	=0.265
	ECL-05	4.96 ± 0.194	
Third Instar	Harlan	5.02 ± 0.165	≤0.001 *
	ECL-05	4.52 ± 0.119	
Fourth Instar	Harlan	5.42 ± 0.190	≤0.001 *
	ECL-05	4.74 ± 0.124	
Fifth Instar	Harlan	7.36 ± 0.284	≤0.001 *
	ECL-05	6.30 ± 0.146	
Adult	Harlan	173.38 ± 7.498	=0.762
	ECL-05	176.02 ± 8.079	

* Means followed by a star indicate significant statistical difference (*p* ≤ 0.05).

### 3.2. Abridged Cohort Table of Rabbit Blood Fed Bed Bugs

Rabbit blood-fed bed bugs had lower life expectancies. HS and ECL-05 strain bed bugs reared on rabbit blood have a life expectancy (*e_x_*) of approximately 149 and 174 days respectively (Table 4, Table 5). While HS bed bugs reared on rabbit blood have an 88% chance of surviving to adulthood, and are expected to live an additional 115 days once reaching the adult stage, ECL-05 bed bugs have a higher chance of reaching the adult stage (94%), and are expected to live an additional 147 days once reaching the adult stage. 

**Table 4:** Stage specific life table of Harlan strain *Cimex lectularius* on rabbit blood.

Harlan Strain reared on Rabbit Blood									
Stage (n = duration in days)	*N_x_*	Age Interval (*x*)	(*l_x_*)	(*_n_p_x_*)	(*_n_q_x_*)	(*_n_d_x_*)	(*_n_L_x_*)	(*T_x_*)	(*e_x_*)
Egg (n = 5)	50	0–5	1.00	1.00	0.00	0.00	5.00	149.49	149.49
First Instar (n = 8)	50	5–13	1.00	0.96	0.04	0.04	7.84	144.49	144.49
Second Instar (n = 8)	48	13–21	0.96	0.96	0.04	0.04	7.52	136.65	142.34
Third Instar (n = 9)	46	21–30	0.92	0.98	0.02	0.02	8.19	129.13	140.36
Fourth Instar (n = 10)	45	30–40	0.90	0.98	0.02	0.02	8.90	120.94	134.38
Fifth Instar	44	40–52	0.88	1.00	0.00	0.00	10.56	112.04	127.32
Adult (n = 40)	44	52–92	0.88	0.98	0.02	0.02	34.80	101.48	115.32
Adult (n = 40)	43	92–132	0.86	0.77	0.23	0.20	30.40	66.68	77.53
Adult (n = 40)	33	132–172	0.66	0.58	0.42	0.28	20.80	36.28	54.97
Adult (n = 40)	19	172–212	0.38	0.42	0.58	0.22	10.80	15.48	40.74
Adult (n = 30)	8	212–242	0.16	0.75	0.25	0.04	4.20	4.68	29.25
Adult (n = 8)	6	242–250	0.12	0.00	1.00	0.12	0.48	0.48	4.00

* Notes: *x* is age interval in days, *N* is the number of individuals alive at the beginning of age *x*, *l_x_* is the probability of surviving to age *x*, *_n_q_x_* is the proportion of individuals alive at the onset of the interval, age x that die prior to reaching the end of the interval (*x* + *n*) where n is the duration spent in the interval, *_n_d_x_* is the fraction of the original cohort that die between the interval (*x* to *x* + *n*), *_n_L_x_* is the fraction of interval lived during the designated interval (*x* to *x* + *n*) per individual, *T_x_* is the total number of days lived beyond age (*x*), and *e_x_* is the life expectancy of an individual alive at age *x*.

**Table 5:** Stage specific life table of ECL-05 strain *Cimex lectularius* on rabbit blood.

ECL-05 reared on Rabbit Blood									
Stage (n = duration in days)	*N_x_*	Age Interval (*x*)	(*l_x_*)	(*_n_p_x_*)	(*_n_q_x_*)	(*_n_d_x_*)	(*_n_L_x_*)	(*T_x_*)	(*e_x_*)
Egg (n = 6)	50	0–6	1.00	1.00	0.00	0.00	6.00	173.63	173.63
First Instar (n = 5)	50	6–11	1.00	0.98	0.02	0.02	4.95	167.63	167.63
Second Instar (n = 5)	49	11–16	0.98	0.98	0.02	0.02	4.85	162.68	166.00
Third Instar (n = 5)	48	16–21	0.96	1.00	0.00	0.00	4.80	157.83	164.41
Fourth Instar (n = 7)	48	21–28	0.96	1.00	0.00	0.00	6.72	153.03	159.41
Fifth Instar (n = 9)	48	28–37	0.96	0.98	0.02	0.02	8.55	146.31	152.41
Adult (n = 40)	47	37–77	0.94	0.89	0.11	0.10	35.60	137.76	146.55
Adult (n = 40)	43	77–117	0.84	1.00	0.00	0.00	33.60	102.16	121.62
Adult (n = 40)	43	117–157	0.84	0.95	0.05	0.04	32.80	68.56	81.62
Adult (n = 40)	40	157–197	0.80	0.55	0.45	0.36	24.80	35.76	44.70
Adult (n = 40)	22	197–237	0.44	0.18	0.82	0.36	10.40	10.96	24.91
Adult (n = 14)	4	237–251	0.08	0.00	1.00	0.08	0.56	0.56	7.00

* Notes: *x* is age interval in days, *N* is the number of individuals alive at the beginning of age *x*, *l_x_* is the probability of surviving to age *x*, *_n_q_x_* is the proportion of individuals alive at the onset of the interval, age x that die prior to reaching the end of the interval (*x* + *n*) where n is the duration spent in the interval, *_n_d_x_* is the fraction of the original cohort that die between the interval (*x* to *x* + *n*), *_n_L_x_* is the fraction of interval lived during the designated interval (*x* to *x* + *n*) per individual, *T_x_* is the total number of days lived beyond age (*x*), and *e_x_* is the life expectancy of an individual alive at age *x*.

Significant differences were found in all life stages when comparing the mean time rabbit blood fed HS and ECL-05 bed bugs spent in each instar. While the ECL-05 strain remained in the egg stage significantly longer than the HS, HS bed bugs required more development time in all instars when reared on rabbit blood (Table 6). When evaluating the total life span of HS and ECL-05 bed bugs reared on rabbit blood, ECL-05 survived 174.02 ± 7.379 days, which was significantly longer than HS (153.28 ± 8.848 days, *p* = 0.054).

**Table 6:** Mean survival time (in days) of rabbit blood reared Harlan and ECL-05 strain bed bugs.

Life Stage	Strain	Mean ± S.E.	Mann-Whitney U	*p*
Egg	Harlan	5.10 ± 0.082	407.00	≤0.001 *
	ECL-05	5.88 ± 0.046		
First Instar	Harlan	8.26 ± 0.811	751.50	≤0.001 *
	ECL-05	4.84 ± 0.214		
Second Instar	Harlan	8.06 ± 0.867	787.00	≤0.001 *
	ECL-05	4.54 ± 0.091		
Third Instar	Harlan	8.28 ± 0.915	831.50	=0.003 *
	ECL-05	5.10 ± 0.415		
Fourth Instar	Harlan	8.56 ± 0.915	967.00	=0.050 *
	ECL-05	6.78 ± 0.641		
Fifth Instar	Harlan	10.84 ± 1.018	952.50	=0.040 *
	ECL-05	8.74 ± 0.771		
Adult	Harlan	104.18 ± 8.580	792.50	=0.002 *
	ECL-05	138.14 ± 7.372		
Total Life Span	Harlan	153.28 ± 8.848	971.00	=0.054 *
	ECL-05	174.02 ± 7.379		

* Indicates significant statistical difference (*p* ≤ 0.05).

### 3.3. Survival Based on Blood Regimen

When comparing the development of HS and ECL-05 bed bugs reared on human or rabbit blood, each strain illustrated significant differences in development time during certain instars. For HS bed bugs, individuals fed rabbit blood spent significantly longer amounts of time in the egg stage, third, fourth, and fifth instars. For ECL-05 bed bugs, individuals reared on rabbit blood also spent significantly longer in the egg stage, fourth, and fifth instars. Comparisons of both strains fed on either human or rabbit blood are shown in Table 7.

**Table 7:** Differences in Harlan and ECL-05 development from egg to fifth instar.

Strain		Egg	First Instar	Second Instar	Third Instar	Fourth Instar	Fifth Instar
Harlan	Mann-Whitney U	308.50	987.50	972.50	929.00	755.00	798.50
	*p*	0.000 *	0.064	0.051	0.024 *	0.001 *	0.002 *
ECL-05	Mann-Whitney U	565.00	1054.00	1045.00	1202.50	894.50	681.00
	*p*	0.000 *	0.155	0.127	0.714	0.011 *	0.000 *

* Indicates significant statistical difference (*p* ≤ 0.05).

HS and ECL-05 bed bugs reared on human blood and ECL-05 bed bugs reared on rabbit blood developed significantly faster than HS bed bugs reared on rabbit blood (Table 8). Human blood fed HS bed bugs had a mean survival time of 34.90 ± 5.428 days between the egg stage to the adult stage, which was significantly shorter than the mean development time of HS bed bugs fed on rabbit blood at 49.10 ± 16.884 days (Chi-square = 34.600, *p* ≤ 0.001). ECL-05 strain bed bugs reared on human blood had a mean survival time of 32.10 ± 3.638 days, which was significantly shorter than the mean survival time of bed bugs of the same strain reared on rabbit blood (35.88 ± 9.460 days, Chi-square = 15.988, *p* ≤ 0.001).

**Table 8:** Mean development time (in days) of (laboratory strain) HS and ECL-05 bed bugs reared on two different blood regimens.

Life Stage	Blood Regimen	Strain	Mean ± S.E.
Egg to Fifth Instar Development	Human	Harlan	34.90 ± 5.428 (B)
		ECL-05	32.10 ± 3.638 (B)
	Rabbit	Harlan	49.10 ± 16.884 (A)
		ECL-05	35.88 ± 9.460 (B)

Different letters in parentheses indicate significant statistical difference (*p* ≤ 0.05).

### 3.4. Survival Based on Sex

Strain and blood regimen appear to have an effect on bed bug survival when comparing the two sexes. The survival of HS males reared on human blood was 204.20 ± 9.270 days compared to 218.03 ± 9.614 days for females, which was not statistically significant (Chi-square = 1.468, *p* = 0.226). However, when comparing the mean survival time of males and females of the ECL-05 reared on human blood, the males (223.39 ± 9.90 days) survived significantly longer than the females (188.68 ± 12.456 days, Chi-square = 3.990, *p* = 0.046).

The mean survival of HS female bed bugs reared on rabbit blood (183.26 ± 11.072 days) was significantly longer than the survival of males (158.84 ± 8.881 days) of the same strain (Chi-square = 4.088, *p* = 0.043). However, there were no significant differences (Chi-square = 2.678, *p* = 0.102) when comparing the survival of ECL-05 males and females reared on rabbit blood. Males and females of ECL-05 lived 168.59 ± 9.776 days and 190.731 days respectively.

### 3.5. Survival Based on Mating Status

Neither males nor females reared on rabbit blood were mated. Because bed bugs reared on human blood were mated and those reared on rabbit blood were not, total life span comparisons of HS and ECL-05 bed bugs reared on human and rabbit blood were made only using unmated HS and ECL-05 bed bugs. Mann-Whitney U analyses of unmated bed bugs revealed that when comparing the total life span of unmated bed bugs, HS bed bugs reared on human blood survived 241.00 ± 17.489 days, which was significantly longer than the survival of rabbit blood reared HS bed bugs (153.28 ± 8.848 days, Mann-Whitney U = 63.000, *p* ≤ 0.001). Similarly, when comparing ECL-05 bed bugs reared on human and rabbit blood, human blood fed bed bugs survived 258.57 ± 7.678 days, which was significantly longer than the survival of rabbit blood fed bed bugs (174.02 ± 7.379 days, Mann-Whitney U = 6.5, *p* ≤ 0.001).

## 4. Discussion

The survival of *Cimex lectularius* is greatly influenced by diet, sex, and mating status [25]. The current study illustrates that survivorship of individuals of two strains was not significantly different based on strain, but was significantly impacted on by blood regimen. HS and ECL-05 bed bugs did have differences in developmental time when assessed based on individual instar, but these differences were not present when evaluating the total life span regardless of the blood type used. The most dramatic differences were present when comparing HS bed bugs reared on human blood to those reared on rabbit blood; individuals reared on human blood survived significantly longer than those fed on rabbit blood. Similarly, ECL-05 bed bugs reared on human blood survived significantly longer than their counterparts reared on rabbit blood. This suggests that humans may be a superior host than blood from other animals.

Host choice of hematophagous insects may be the result of various physiological factors [28]. Therefore, once an insect becomes closely associated with a narrow range of hosts, physiological specialization may limit the range of hosts an insect can exploit [40]. While we cannot say for certain, the blood of different species, *i.e.*, guinea pig, chicken, and rabbit, may be deficient in various nutrients compared to human blood. The two blood sources used in this experiment had certain technical and biochemical differences. Bed bugs reared on human blood were allowed to feed on a live host whereas bed bugs reared on a rabbit blood were fed defibrinated rabbit blood via an artificial feeder [22]. Other researchers have illustrated that there are limited tradeoffs between using an artificial feeding system and a live animal. In a paper outlining a novel feeding system for mosquito rearing, Deng *et al.* [41] demonstrated blood feeding on an artificial feeder was not significantly different from blood feeding on live guinea pigs. There was no difference in the fecundity, survival, or the hatchability of eggs reared on a membrane feeding system compared to mosquitoes reared on a live animal. Data derived from *Anopheles* standard research protocols suggests that glass-feeding apparatus is just as effective as animal feeding [42,43]. Additionally, defibrinated rabbit blood is commonly used by bed bug researchers across the United States. However, defibrinated blood may lack certain nutrients normally acquired from a living rabbit host.

Blood feeding insects often use host-related kairomones to locate their hosts [28]. Therefore, bed bugs feeding on a live human host may have been prompted to feed by chemical phagostiumulants which were not present when using artificial feeders for rabbit blood reared bed bugs. In order to avoid this, Parafilm^®^ placed over the artificial feeders in the rabbit blood feeding treatment were rubbed over the skin of a human participant. Despite perceived conflicts in the methodology, results suggest that the use of rabbit blood results in a reduction in bug survival.

Over time, natural selection will maximize the insect’s ability to persist on its major host, which may limit its ability to thrive on other, dissimilar hosts resulting in a shorter lifespan and reduced fitness. Some physiological effects may be detrimental but less apparent than outright mortality; these include reduced developmental rates, reduced food intake and digestion, imbalanced sex ratio, and reduced longevity [28].

Bed bugs exhibited clear differences in development based on both strain and blood source. While there were no significant differences in the sex ratio of HS or ECL-05 reared on human or rabbit blood in this study, other developmental differences were present. Both HS and ECL-05 bed bugs in this study had a reduced rate of development in certain instars. Rabbit blood reared HS bed bugs spent 8.06 ± 0.867 days in the second instar compared to 5.14 ± 0.206 days for HS reared on human blood. Significant differences were also found when comparing rabbit blood and human blood fed bed bugs of the HS during the third, fourth, and fifth instars. Similar results were derived when comparing ECL-05 bed bugs reared on human or rabbit blood. Rabbit blood reared ECL-05 bed bugs spent significantly less time in the fourth and fifth instars compared to those reared on human blood.

Time spent in instar may also be influenced by reduced food intake and digestion. Reduced food intake and reduced digestion rate can result in reduced fecundity in insects [31]. Personal observation indicates that bed bugs were more reluctant to feed on the blood of rabbits. As the methodology illustrates, bed bugs were offered a blood meal every second day to ensure that blood availability was not a limiting factor. However, many bed bugs on the rabbit blood regimen were reluctant to feed. Many would imbibe small amounts of the blood without feeding to repletion. These findings directly contradict previously published research on bed bug survival based on blood regimen. Johnson (1937) investigated the effect of host type on development rate; development rate was the shortest when bed bugs were reared on chicken blood, approximately equal when reared on the blood of pigeon, bat, and rabbit blood, but was the longest when reared on human blood [27]. However, in the present study, bed bug development on rabbit blood is significantly longer than development on human blood. 

There is a threshold amount of blood nutrients required to transition from instar to instar. While in nature a single large meal between molts is more usual, cimicids will still molt after a succession of small meals [44]. Similarly, different host species may prompt blood-feeding insects to obtain blood meals of different sizes based on the presence or absence of essential nutrients like vitamin B and calcium [24,28,45], and protein content [28]. Reduced feeding may suggest that rabbit blood is inferior to the blood of humans.

Bed bugs used in the current study illustrate differing longevity based on sex. The HS bed bugs reared on human blood, had no significant differences in male or female survival. However, in the ECL-05 bed bugs reared on human blood, male survival was significantly longer than female survival. This finding is not particularly surprising as there are certain costs associated with traumatic insemination. The act of copulation can reduce female survival by up to 30% [15]. Despite the evolution of the spermalege as a counter adaptation to traumatic insemination [46,47], repeated wounding attributed to multiple mating events can reduce female survival by up to 50% [15]. Nevertheless, it seems that this may not be the case for all treatments considered in the current experiment. While ECL-05 bed bugs reared on rabbit blood illustrated no significant differences based on sex, HS females survived longer than HS males reared on rabbit blood. 

While it is clear that bed bugs can be reared, the blood of secondary hosts like chickens, guinea pigs, and in this case rabbits, the current study demonstrates that there are negative developmental effects of doing so. Much of the current bed bug research is derived from experiments rearing bed bugs on alternative blood sources. Currently, we are still unaware of any behavioral effects of bed bug rearing on alternative hosts. However, the combination of poor diet and genetic make-up based on strain has the potential to influence research results. When comparing the survival of unmated HS bed bugs, rabbit blood reared bed bugs experienced a 36.4% reduction in survival compared to human blood reared bed bugs. This factor can become extremely important in other research where bed bug survival is contingent on exposure to a treatment, particularly survival following pesticide exposure [20,48,49,50]. Physiological changes brought on by poor diet can affect control strategies such as pesticide or biopesticide application [51]. Therefore, the current study suggests that research results should take bed bug feeding regimen into account when interpreting results. 

Bed bug management may also be affected. Inspections, treatments and retreatments often rely on the ability to accurately predict population changes and reproductive rates. Inaccurate predictions prompted by laboratory assessments using suboptimal blood hosts can impair management decisions.

## 5. Conclusions

Bed bug development and ultimate survival can be influenced by many factors including temperature, nutritional status of the blood meal, duration of the blood meal and amount of blood imbibed, sex, and mating status [8,25,27,47]. This study was the first step to evaluating the effect of feeding regimen on bed bug growth, development, and survival. Though it is not clear what the particular differences between the blood sources are, results illustrate that rabbit blood appears to be an inferior blood source compared to human blood, regardless of strain. Additionally, ECL-05 strain tends to have a higher survival rate than HS, which has been lab reared for more than 30 years. Still, there are many other factors that could have played an important role in the survival of bed bug populations, and those factors should be investigated further in the future. Future studies should continue to evaluate the effect of blood regimen on bed bug growth and development. Additional factors to be tested should include the effect of bed bug feeding on a live host *versus* an artificial feeding system. Future studies should also expand to include blood from other animals commonly used in bed bug research including chicken and guinea pigs. Additionally, future studies should elucidate the biochemical and nutritional differences between the various blood sources. Knowledge of bed bug development and longevity can assist in the development of safe, effective, and practical control measures.
